# The fungus *Kalmusia longispora* is able to cause vascular necrosis on *Vitis vinifera*

**DOI:** 10.1371/journal.pone.0258043

**Published:** 2021-10-15

**Authors:** Zoltán Karácsony, Dániel G. Knapp, Szabina Lengyel, Gábor M. Kovács, Kálmán Zoltán Váczy

**Affiliations:** 1 Food and Wine Research Institute, Eszterházy Károly University, Eger, Hungary; 2 Department of Plant Anatomy, Institute of Biology, Eötvös Loránd University, Budapest, Hungary; 3 Plant Protection Institute, Centre for Agricultural Research (ATK), Eötvös Loránd Research Network (ELKH), Budapest, Hungary; Leibniz-Institut fur Naturstoff-Forschung und Infektionsbiologie eV Hans-Knoll-Institut, GERMANY

## Abstract

Fungal diseases in agronomically important plants such as grapevines result in significantly reduced production, pecuniary losses, and increased use of environmentally damaging chemicals. Beside the well-known diseases, there is an increased interest in wood-colonizing fungal pathogens that infect the woody tissues of grapevines. In 2015, a traditional isolation method was performed on grapevine trunks showing symptoms of trunk diseases in Hungary. One isolate (T15142) was identified as *Kalmusia longispora* (formerly *Dendrothyrium longisporum*) according to morphological and phylogenetic analyses. To evaluate the pathogenicity of this fungus on grapevines, artificial infections were carried out under greenhouse and field conditions, including the CBS 824.84 and ex-type CBS 582.83 strains. All isolates could be re-isolated from inoculated plants; however, varying virulence was observed among them in terms of the vascular necrosis caused. The incidence and severity of this symptom seemed to be congruent with the laccase-producing capabilities of the isolates. This is the first report on the ability of *Kalmusia longispora* to cause symptoms on grapevines, and on its possible dependence on laccase secretion.

## Introduction

*Vitis vinifera* L. is one of the most important perennial crops in agriculture, with high economic, cultural, and touristic impacts. The major threats to the sustainable management of grapevine plantations are the pests and diseases that severely affect the condition of the plants, as well as the yield and quality of the berries. Studies about the connection between climate and the distribution of the pests and pathogens of grapevines suggest that changes in the climate will create novel challenges for grape growers [1–3]. Fungal infections account for many of the diseases observed in grapevines [4]. Beside the well-known diseases such as powdery mildew and downy mildew and grey and black rot, vascular pathogens are receiving growing attention. These latter fungi cause the so called grapevine trunk diseases (GTDs). This group consist of Blackfoot disease, Botryosphaeria dieback, Eutypa dieback, Esca disease and Phomopsis disease [5]. Several fungal taxa are known to be associated with these diseases and their number is increasing rapidly. The characteristic symptoms are the necrotic lesions in the wood, the impaired morphology of green shoots and the discoloration and necrosis of leaves. In its severe form, Esca disease can even cause the sudden death of the infected plant called apoplexy [5]. The management of GTD-associated pathogens is a great challenge for vine-growers. Since the ban of sodium arsenite there is no efficient chemical control against these fungi [6]. The application of well-known and satisfactory management procedures is hindered by several factors. The widely-used fungicidal sprays cannot reach the pathogens, which colonize the inner tissues of the trunks [7]. The unsatisfactory knowledge about the host-pathogen-environment interactions makes the development of alternative control methods against GTDs difficult. All the above-mentioned problems indicate that adequate information need to be collected about the causal agents of GTDs for the development of efficient management techniques.

The ascomycetous genus *Kalmusia* was established nearly 150 years ago [8] by the type species *Kalmusia ebuli*, and formerly consisted of more than 40 filamentous fungal species [9]. They are common members of the microbiome of various plants and their host range is wide; these fungi can be obtained from, for example, bamboo [10], oak [11], and raspberry [12]. The genus has been truncated, and nowadays, *Kalmusia sensu stricto* comprises less than 10 species [13,14]. In 2014, Verkley et al. [15] described the species *Dendrothyrium longisporum*, later assigned to the *Kalmusia* genus [16]. The morphological and molecular characterization of the original species was based on two strains, which were isolated from dwarf mistletoe (*Arceuthobium pusillum*) and common wheat (*Triticum aestivum*) [15]. Our knowledge about the lifestyle of the fungus is limited. Despite the fact that it seems to occur in plant material, the possible connection between *Kalmusia longispora* and plant diseases had not been examined before this study. A close relative of *K*. *longispora* is *Kalmusia variispora* (formerly *Dendrothyrium variisporum*); the strains used for the description of *K*. *variispora* (CBS 121517 and CBS 197.82) were isolated from a declined grapevine in Syria and from the dicotyledonous plant winter heath (*Erica carnea*) in Switzerland [15]. In addition, *K*. *variispora* was also reported on Persian oak (*Quercus brantii*) in Iran [11] and on symptomatic grapevine trunks in Croatia [17]. Recently, the pathogenicity of *K*. *variispora* was confirmed on grapevines [18]. It was shown that *K*. *variispora* isolates can develop necrotic lesions in the woody tissues of grapevine cuttings. Except for this study, we are not aware of any other confirmed case of phytopathogenicity in *K*. *sensu stricto*.

During the isolation-based examination of the mycobiota of grapevines, an isolate was found to be *K*. *longispora*, originating from a plant with symptoms of trunk diseases. The aims of the present work were to verify and confirm the identity of the isolate and to examine of the capability of *K*. *longispora* isolates to develop symptoms on grapevines.

## Results

### Identification and phylogenetic analysis of the T15142 isolate

**Fig 1:**
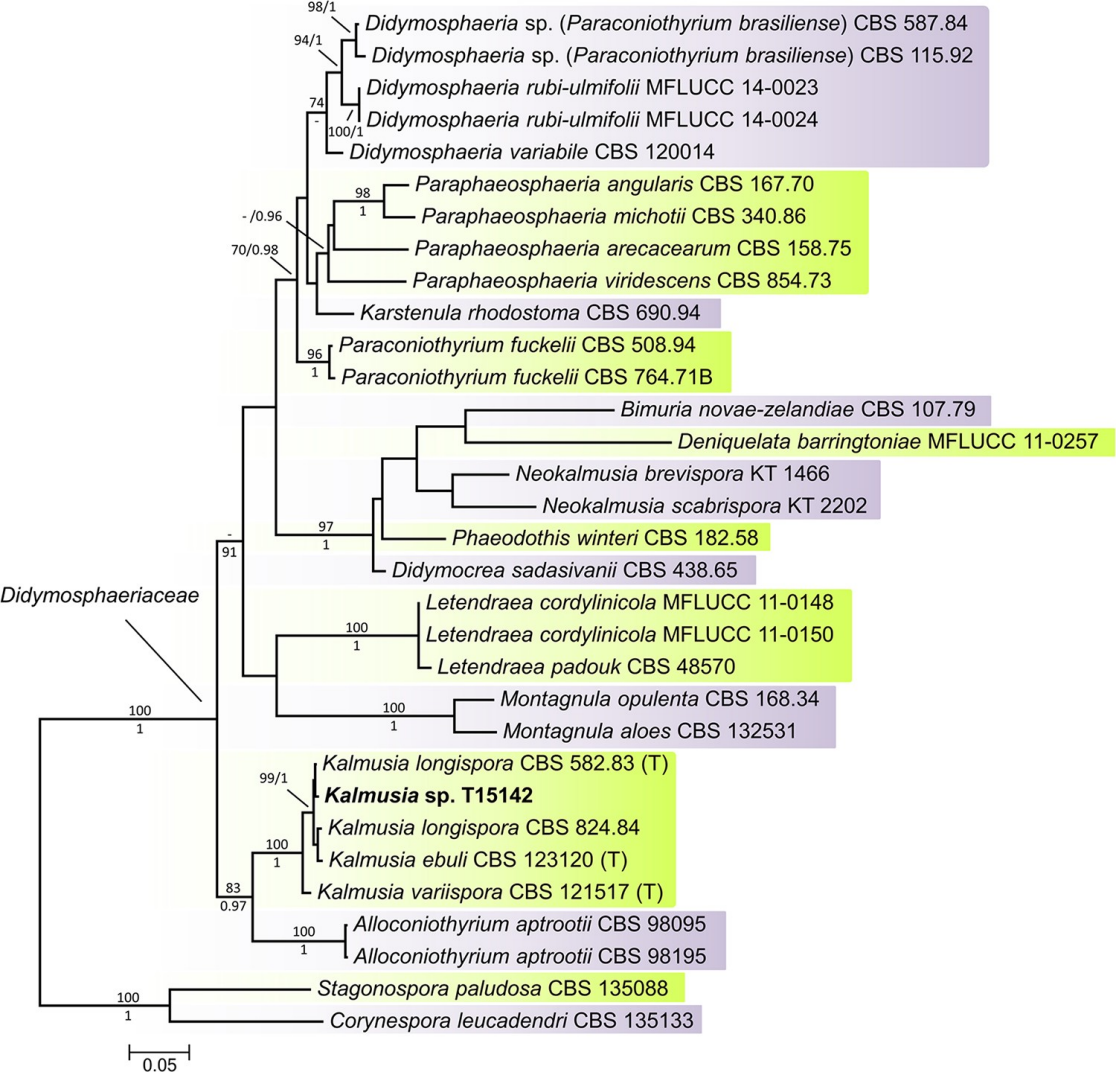
Maximum likelihood (RAxML) tree of concatenated internal transcribed spacer (ITS), partial large ribosomal subunit (LSU), and partial β-tubulin (TUB) sequences of the representative species of *Didymosphaeriaceae*. Bayesian posterior probabilities (≥0.90) are shown below branches and after slashes; RAxML bootstrap support values (≥70) are shown above branches and before slashes. The strain introduced in this study is shown in bold. *Corynespora leucadendri* and *Stagonospora paludosa* served as outgroups. The *Kalmusia* genus type materials (T) are indicated. The scale bar indicates the expected changes per site per branch.

During the collection of the isolate from the Kékfrankos grapevine variety from Helesfa, Pécs Wine Region, Hungary, one isolate (T15142) showed high internal transcribed spacer (ITS) sequence similarity with *K*. *longispora* strains using a BLAST search in GenBank. Based on the results of the phylogenetic analysis of the ITS, partial large ribosomal subunit (LSU), and partial β-tubulin (TUB), the T15142 isolate belongs to the pleosporalean family *Didymosphaeriaceae* and forms a well-supported clade with *Kalmusia* species, a sister group of *Alloconiothytium aprootii* (Fig 1). Isolates of *K*. *longispora* and *K*. *ebuli* grouped together within the genus and T15142 showed the highest similarities with the two *K*. *longispora* strains. The ITS, LSU, and TUB sequences of this isolate showed 99.2%, 100%, and 99.8% similarity with the *K*. *longispora* ex-type strain CBS 582.83, respectively [15]. The results of the molecular phylogenetic analysis unambiguously show that the isolate T15142 represents *K*. *longispora*.

### Morphological characterization of the T15142 isolate

**Fig 2:**
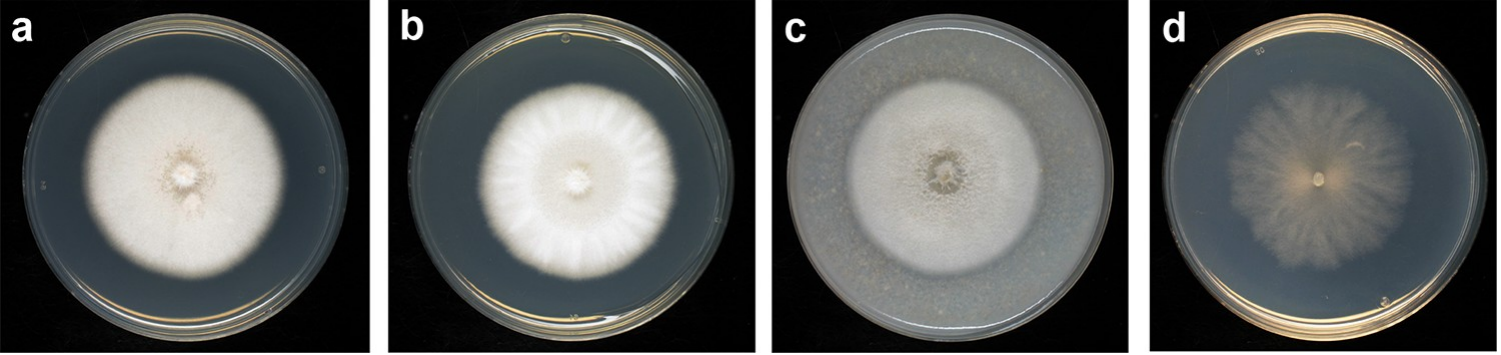
Two-week-old colonies of the *K*. *longispora* isolate T15142 from Hungary. The colonies were grown at 25°C on different media: (**a**) Potato dextrose agar (PDA); (**b**) malt extract agar (MEA); (**c**) oatmeal agar (OA); (**d**) water agar (WA).

**Fig 3:**
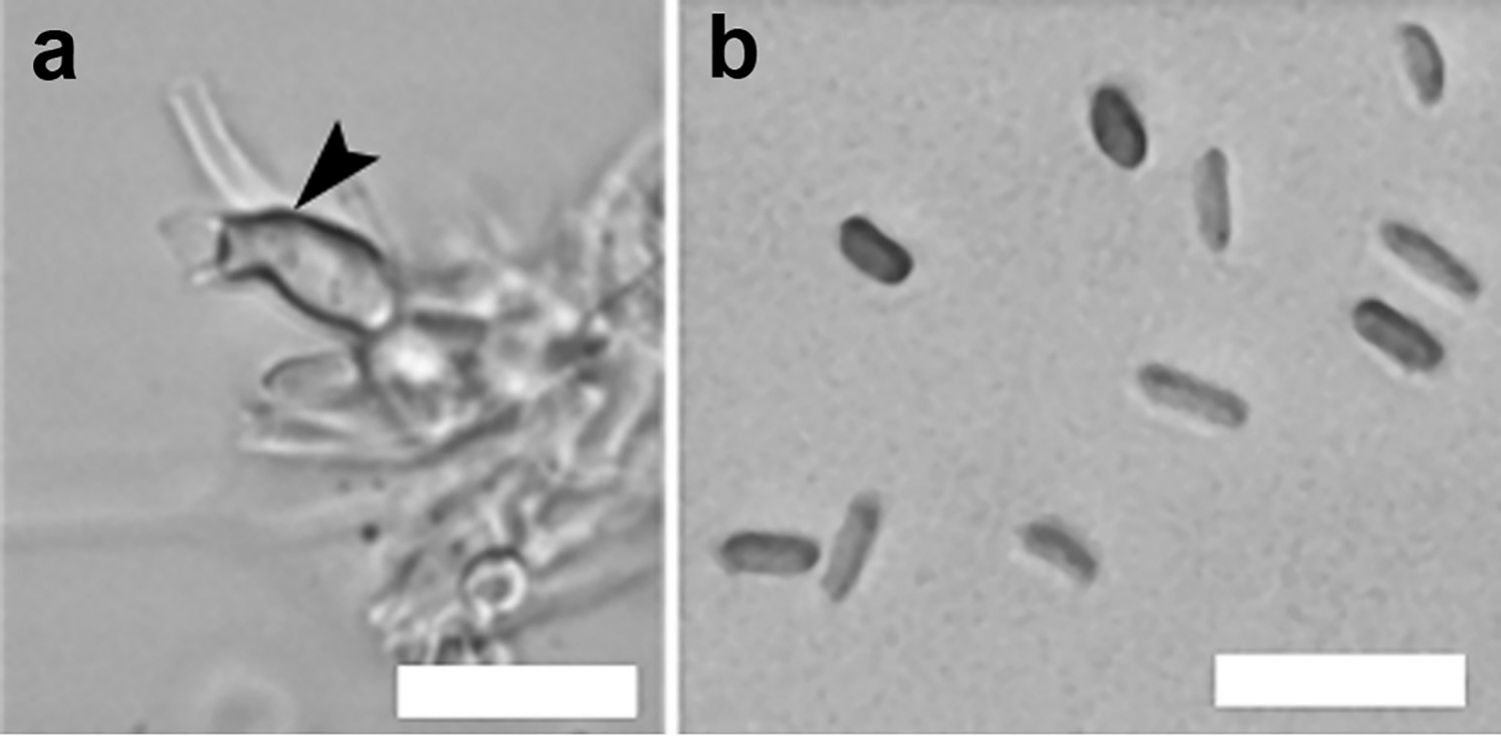
Reproductive cells of the *K*. *longispora* isolate T15142. Sporulation was induced on pine needles placed on PDA medium. (**a**) Conidiogenous cell (arrowhead); (**b**) conidia. Scale bars = 10 μm.

To further characterize the T15142 strain, micro- and macromorphological examinations were performed. Colonies of the isolate were white with entire margins on potato dextrose agar (PDA), malt extract agar (MEA), and oatmeal agar (OA) media, while developed a pale-brownish color with undulated margins on water agar (WA) medium (Fig 2). The conidia of T15142 are cylindrical ellipsoids with dimensions of 4.21 ± 0.3 × 1.74 ± 0.72 μm (Fig 3B). Conidiogenous cells were also observed in the disrupted pycnidia (Fig 3A).

### Pathogenicity tests of the *K*. *longispora* isolates on grapevines

**Fig 4:**
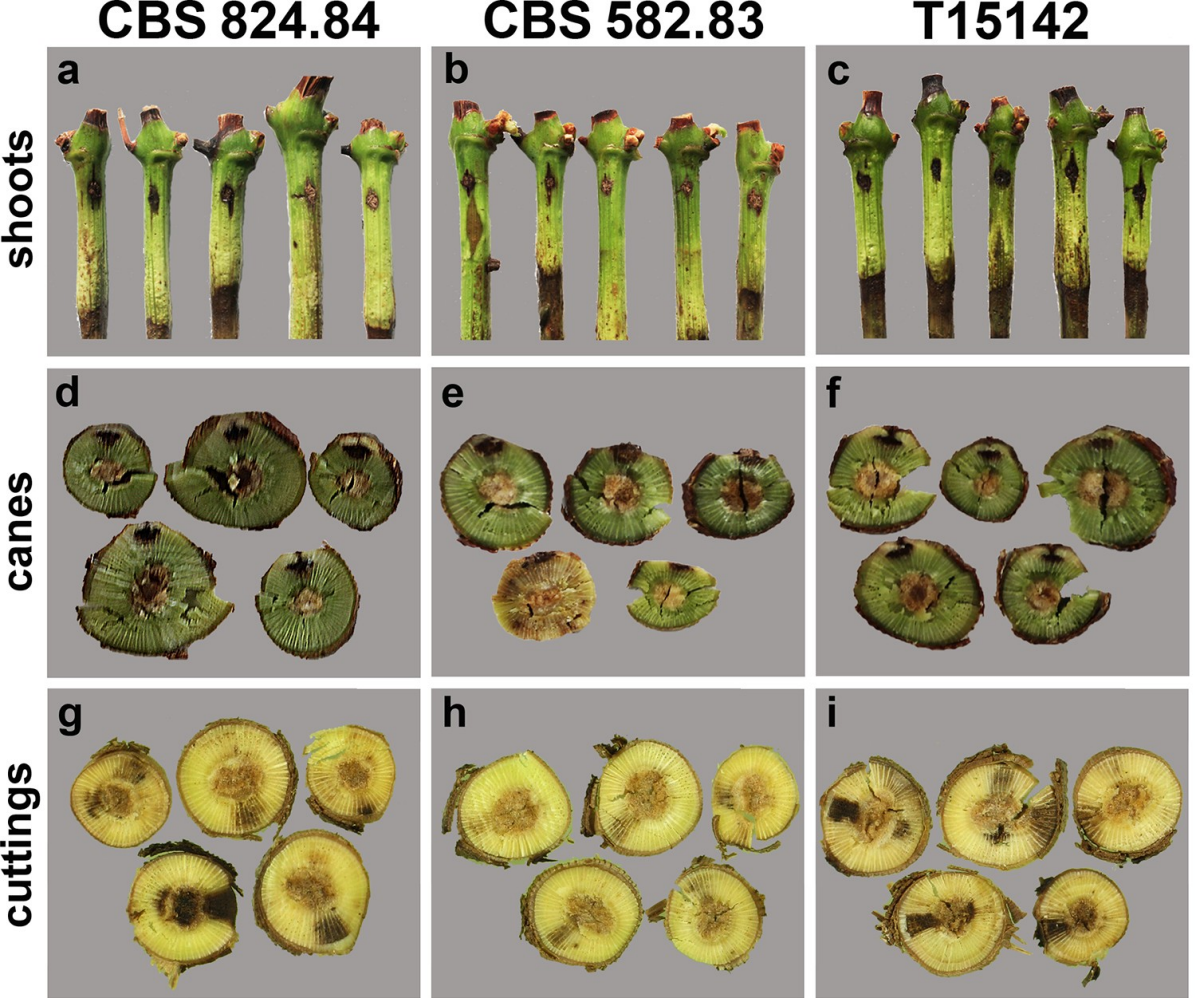
Artificial infection of *V*. *vinifera* cv. Cabernet Sauvignon with *K*. *longispora* isolates. (**a–c**) Shoots 21 days after inoculation with CBS 824.84 (**a**), CBS 582.83 (**b**), or T15142 (**c**) *K*. *longispora* strains. (**d–f**) Cross-sections of canes 90 days after inoculation with CBS 824.84 (**d**), CBS 582.83 (**e**), or T15142 (**f**) strains. (**g–i**) Cross-sections of cuttings 80 days after inoculation with CBS 824.84 (**g**), CBS 582.83 (**h**), or T15142 (**i**) strains.

Artificial infections were performed to examine the pathogenicity of the *K*. *longispora* strains on grapevines. All three of the tested strains caused different incidences of symptoms on the shoots (Fig 4A–4C). The isolate T15142 produced longitudinal, black necrotic lesions on all five infected shoots (Fig 4C), while the CBS 824.84 and CBS 582.83 strains developed necrosis on three and two shoots, respectively (Fig 4A and 4B). Non-symptomatic shoots showed brownish coloration at the inoculation point. The incidence of symptoms developed on canes (Fig 4D–4F) was somewhat similar. While both CBS 824.84 and T15142 caused black necrosis deep in the xylem on all five inoculated canes (Fig 4D and 4F), strain CBS 582.83 developed only slight surface discoloration at the inoculation point (Fig 4E). Radial necroses can be observed on cross sections of all the infected cuttings (Fig 4G–4I). However, this symptom was much more definite in case of CBS 824.484 (Fig 4G) and T15142 (Fig 4I), while CBS 582.83 caused only mild discolorations (Fig 4H). All of the tested strains were re-isolated frequently from all types of the inoculated plants. The isolate T15142 was identified in four out of the five inoculated shoots and in all five of the canes and cuttings. Both CBS 582.83 and CBS 824.84 could be re-isolated from all the infected shoots and canes and from four of the inoculated cuttings.

### Exoenzyme production of *K*. *longispora* isolates

**Fig 5:**
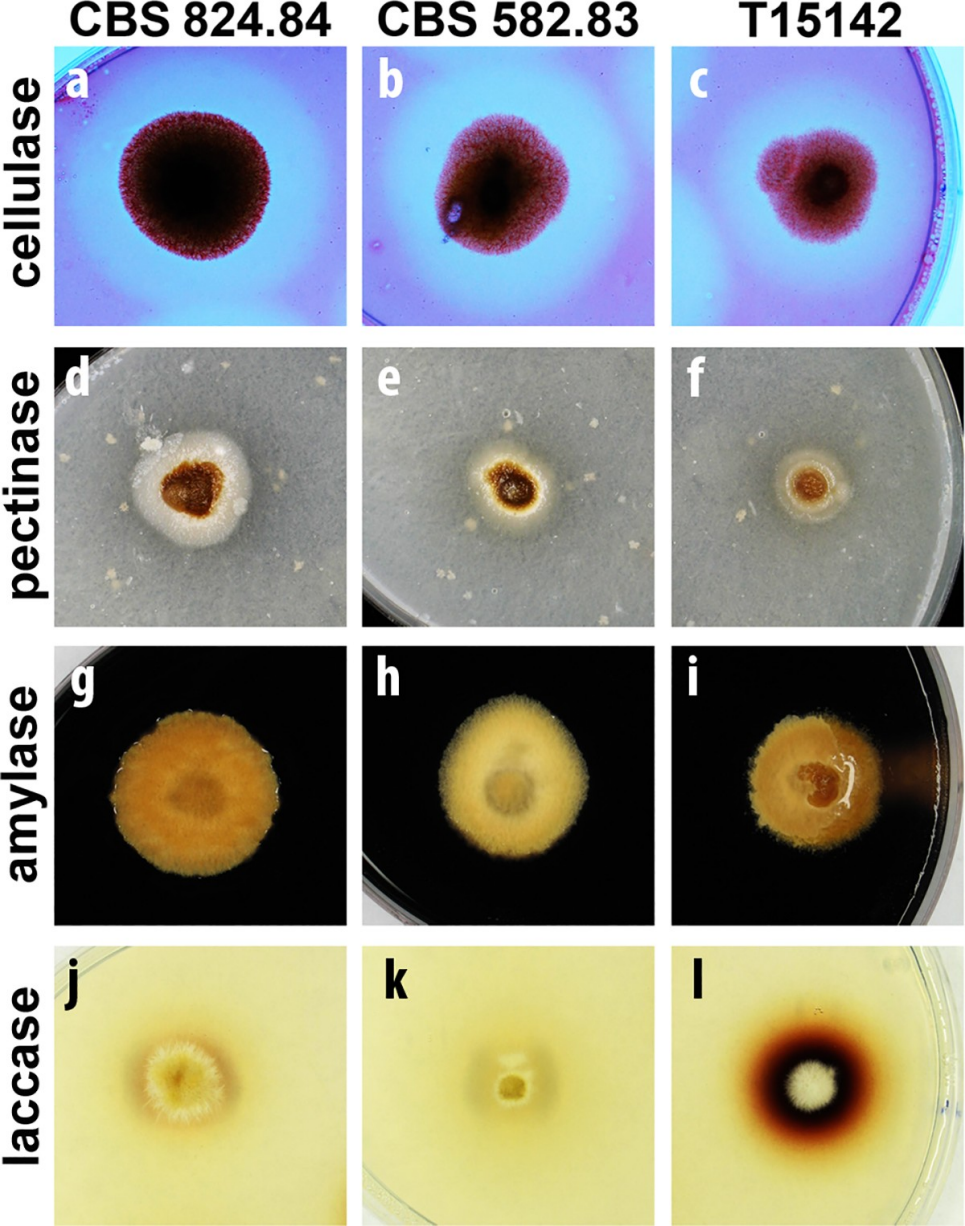
Exoenzyme production of *K*. *longispora* isolates on indicative media. Strains CBS 824.84 (**a**,**d**,**g**,**j**), CBS 582.83 (**b**,**e**,**h**,**k**), and T15142 (**c**,**f**,**i**,**l**) growing on indicative media for cellulases (**a**–**c**), pectinases (**d**–**f**), amylases (**g**–**i**), and laccases (**j**–**l**). Photographs were taken after 11 (**a**–**i**) or 6 days (**j**–**l**) of incubation at 25°C.

Examination of the digestive exoenzymes revealed differences between the *K*. *longispora* strains tested here (Fig 5). All strains showed equally large zones of clearance on cellulose-indicating medium (Fig 5A–5C). A very faint hydrolytic zone could be observed around the colonies of all *K*. *longispora* strains on pectinase-detecting medium (Fig 5D–5F). None of the strains produced amylases according to Lugol’s staining of starch-containing medium, indicated by the absence of a yellowish zone around the colonies (Fig 5G–5I). On guaiacol-amended medium, the T15142 isolate was surrounded with a wide red-brownish halo (Fig 5L), indicating high laccase activity. This zone was very faint and narrow in the case of CBS 824.84 (Fig 5J) and completely absent in the case of CBS 582.83 (Fig 5K).

**Fig 6:**
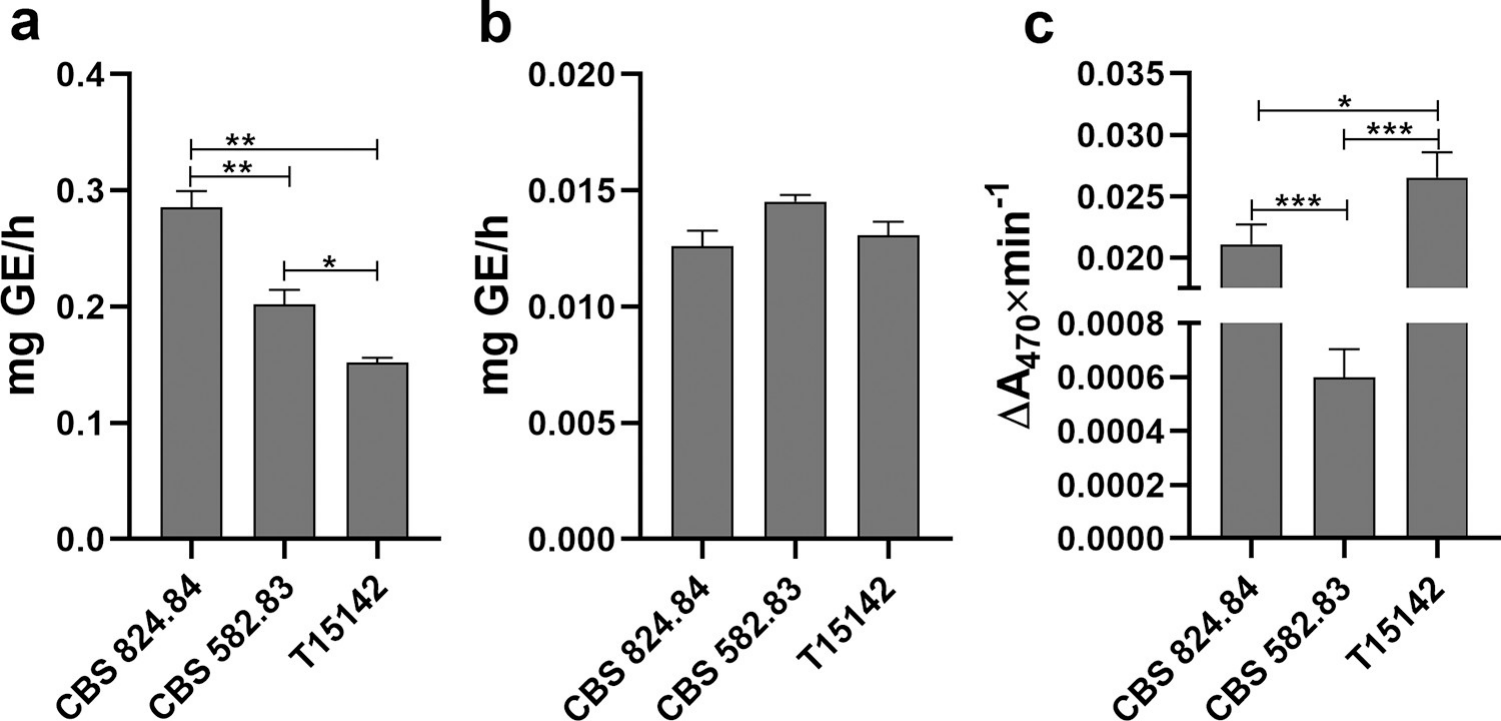
Exoenzyme production of *K*. *longispora* isolates in liquid cultures. Cellulase (**a**) pectinase (**b**) and laccase (**c**) activities of strains CBS 824.84, CBS 582.83, and T15142 grown in liquid minimal media for 7 days at 25°C with 180 rpm shaking. GE: Glucose equivalent of released reducing sugars. Asterisks mark significances of differences (* p<0.05, ** p<0.005, *** p<0.0005).

Comparison of the exoenzyme activities in liquid cultures of the *K*. *longispora* isolates by spectrophotometric methods showed similar results as observed on indicative media (Fig 6, S1 Table). The measured cellulase activities were high and slightly but significantly differed between the strains (Fig 6A). Pectinase activities were very low and no difference could be observed between the examined strains (Fig 6B). The highest laccase activity was measured in the culture filtrate of T15142 followed by a somewhat lower value in the case of CBS 824.484. The activity of laccases secreted by CBS 582.83 was 45 times lower relative to T15142 and 30 times lower than CBS 824.484 (Fig 6C).

## Discussion

Grapevines live together with a wide spectrum of fungi [19]. Recently, numerous studies have focused on the grapevine microbiome, including endophytic, pathogenic, and even saprotrophic fungi using different approaches and techniques [19,20], providing us with important information on the presence of potential pathogens. Although, there are broad-scale studies on considerable grapevine pathogens [21] and continuously increasing information on the fungi involved in trunk diseases [22,23]. In the present study, we introduced *K*. *longispora* as species associated with vascular fungal infections of grapevines. Experiments were carried out with a strain isolated from a grapevine trunk in Hungary, and all of the available strains were deposited into culture collections (CBS 582.83 and CBS 824.84), including the ex-type material on which the description of *K*. *longispora* was carried out [15]. The strain characterized in the present study (T15142) was isolated from the trunk of a symptomatic Cabernet Sauvignon grapevine, a variety highly susceptible to GTDs. The plantation was relatively old (15 years) and showed high incidence (18.6%) of GTD-related symptoms in the year of the isolation. The following well-known causal agents of different GTDs were also isolated from the same trunk: *Eutypa lata* (Eutypa dieback), *Neofusicoccum parvum* (Botryosphaeria dieback), and *Phaeoacremonium minimum* (Esca disease). Therefore, because of the diversity of the co-occurring pathogenic species, this fungus cannot be solely linked to and/or considered a solely causal agent of any of the above-mentioned infections.

The fungus *K*. *longispora* belongs to the family *Didymosphaeriaceae* (formerly *Montagnulaceae*), which includes saprobes, endophytes, and pathogens associated with a wide variety of substrates worldwide [13]. The genus *Kalmusia* represents one of the basal lineages of the family and comprises endophytic or saprobic fungi mainly of the *Santalaceae* and *Poaceae* families [14]. *Kalmusia*s species are reported to have *Coniothyrium*-like, *Cytoplea*, *Microsphaeropsis*, and *Paraconiothyrium* asexual morphs, which was also demonstrated here. The isolate T15142 showed all of the micro- and macromorphological characteristics of *K*. *longispora* described earlier by Verkley et al. [15].

All three *K*. *longispora* strains examined in the present study were originated from phylogenetically distant plant hosts, which fact suggests a general association of this fungus to plants. However, we are not aware of any studies on the possible relation of *K*. *longispora* to any plant disease. According to our results, *K*. *longispora* can cause symptoms on grapevines and its virulence shows high variances among strains. The most virulent strain was T15142 causing severe symptoms an all types of examined grapevine material and the least virulent was CBS 582.83 especially on canes and cuttings. The higher virulence of CBS 824.84 on canes and cutting rather than on the shoots could be explained by the longer incubation time between the inoculation and the observation. The same growing capacity of the tested strains in grapevines regardless of their virulence implies that the lifestyle of this fungus strongly depends on the host. Based on these results, we hypothesize that *K*. *longispora* can potentially act as a pathogen, depending on the strain and/or on the host plant. The pathogenicity tests did not showed correlation between pathogen multiplication and virulence like it was demonstrated earlier in other types of fungal plant pathogens [24,25]. The most virulent T15142 and the least virulent CBS 582.83 strains were both isolated from dicotyledonous woody perennials, while CBS 824.84 with moderate virulence originated from a monocotyledonous herbaceous annual plant. This suggests that the very low virulence of CBS 582.83 is more likely due to the lack or loss of virulence rather than the inadequate adaptation of this strain to grapevines as hosts. Various biological background can result different virulence of conspecific strains. The different origin of the strains propose the role of host-pathogen coevolution [26], however the long- term preservation of the CBS strains should be also taken into account.

The results of the pathogenicity tests imply that some of the virulence factors were possibly synthesized in a lower amount by CBS 824.84 than by T15142, and these factors may be nearly absent in CBS 582.83. The high rate of re-isolation of all strains from the infected grapevines indicates that these virulence factors are responsible for the damage of plant tissues, rather than the growth ability of the fungi inside the plant. Since the wood necrosis in grapevines affected by trunk diseases is caused by the digestive exoenzymes of the pathogens [27–29], we also examined the *K*. *longispora* strains from this perspective. The three *K*. *longispora* strains were proven to be equally potent producers of cellulases, poor producers of pectinases and none of them showed amylolytic activity. The observed differences in the laccase secretion of the isolates seemed to be concurrent with the results of the pathogenicity tests; however, the varying isolation date and the preservation of the three strains under different circumstances may also have an effect on exoenzyme production. The high level of laccases secreted by T15142 could explain the high incidence of symptoms observed on the shoots and the capability of the isolate to necrotize the xylem of canes and cuttings. The moderate laccase activity in the case of CBS 824.84 may have contributed to the lower incidence of symptoms observed on the shoots and the restoration of virulence after a longer time of growing in canes and cuttings. The absence of laccase signal on indicative media and the very low activity measured in liquid cultures in case of CBS 582.83 may explain the low symptom incidence on the shoots and the inability of the strain damage the lignified tissues of canes and cuttings.

Herein, we showed, for the first time, the ability of *K*. *longispora* to cause vascular necrosis on grapevines, as well as the possible importance of laccases in the development of symptoms. This study contributes to the growing knowledge on an agriculturally important group of disease agents, the growing group of GTD-associated pathogens.

## Materials and methods

### Isolation of fungal strains

In 2015, five wine regions in Hungary (i.e., Eger, Neszmély, Pécs, Szekszárd, and Villány) were monitored for grapevines exhibiting symptoms of trunk diseases, and then the wood-colonizing fungi were isolated. Three thin discs were cut from the trunks. The traditional isolation protocol was carried out according to Váczy et al. [23]: The discs were surface-sterilized in 1% chloramine B solution for 5 min after the bark tissues were removed. The samples were rinsed in sterile distilled water and dried. Then, five wood chips were cut and placed on potato dextrose agar plates (PDA; Sigma-Aldrich, Germany). The plates were incubated at room temperature (21 ± 2°C) and the emerging mycelia were transferred to new PDA plates to obtain pure cultures for morphological and molecular works. In this study, three *K*. *longispora* strains, namely, CBS 582.83 (ex-type culture), CBS 824.84, and T15142, from Hungary were examined.

### Phylogenetic analysis

For molecular identification of the chosen strains, DNA was extracted from the lyophilized mycelia grown on cellophane-covered PDA media using a DNeasy Plant Mini Kit (Qiagen, Germany). Polymerase chain reactions (PCRs) were performed to amplify the ITS region with the ITS1F [30] and ITS4 [31] primers. The LSU and TUB genes of this isolate were also amplified and sequenced using the LR0R and LR5 [32] and Btub2Fd and Btub4Rd [33] primer pairs, respectively. Sequences were compiled from electropherograms and edited using BioEdit version 7.1.9 software [34]. The ITS, LSU, and TUB sequences were deposited in NCBI GenBank under the accession numbers MN945157, MN945151, and MN939397, respectively. The isolate was deposited to the CBS-KNAW Culture Collection (Westerdijk Fungal Biodiversity Institute, the Netherlands) under the accession number CBS 144250.

The sequences were aligned with the sequences of respective loci from GenBank (Table 1) by the E-INS-i method of the online MAFFT program version 7 [35]. The alignments were checked and edited in MEGA6 [36]. Multi-locus phylogenetic Bayesian analysis was performed with MrBayes 3.1.2 [37] using the GTR+G nucleotide substitution model implemented for the ITS, LSU, and TUB sequences. Four Markov chains were run for 10,000,000 generations sampled every 1000 generations, with a burn-in value set at 4000 sampled trees. Maximum likelihood (ML) phylogenetic analysis was carried out with raxmlGUI version 1.3 [38] implementation of RAxML [39]. The GTR+G nucleotide substitution model was applied with ML estimation of the base frequencies, and an ML bootstrap analysis with 1000 replicates was conducted to test the support of the branches. The phylogenetic trees were visualized and edited in MEGA6 [36].

**Table 1:** List of the isolates included in the phylogenetic analysis.

Species	Former identification	Isolate	GenBank accession number			Host	References
			LSU	ITS	TUB		
***Alloconiothyrium aptrootii***	*Coniothyrium* sp.	CBS 980.95 ^T^	JX496234	JX496121	JX496460	nd	[15,16]
***Alloconiothyrium aptrootii***	*Coniothyrium* sp.	CBS 981.95	JX496235	JX496122	JX496461	nd	[15,16]
***Bimuria novae-zelandiae***		CBS 107.79	AY016356	nd	nd	nd	[16]
***Corynespora leucadendri***		CBS 135133	KF251654	KF251150	KF252639	nd	[16]
***Deniquela tabarringtoniae***		MFLUCC 110257	KM213997	KM214003	nd	nd	[16]
***Didymocrea sadasivanii***		CBS 438.65	DQ384103	nd	nd	nd	[16]
***Didymosphaeria rubi-ulmifolii***		MFLUCC 140023	KJ436586	KJ436586	KJ939277	nd	[16]
***Didymosphaeria rubi-ulmifolii***		MFLUCC 140024	KJ436585	KJ436585	KJ939276	nd	[16]
***Didymosphaeria* sp.**	*Paraconiothyrium brasiliense*	CBS 587.84	JX496212	JX496099	JX496438	*Vitis vinifera*	[15,16]
***Didymosphaeria* sp.**	*Paraconiothyrium brasiliense*	CBS 115.92	JX496135	JX496022	JX496361	*Olea europaea*	[15,16]
***Didymosphaeria variabile***	*Paraconiothyrium variable*	CBS 120014	JX496139	JX496026	JX496365	*Actinidia chinensis*	[15,16]
***Kalmusia ebuli***		CBS 123120	JN644073	nd	nd	nd	[16]
***Kalmusia italica***		MFLUCC 130066 ^T^	KP325441	KP325440	nd	*Spartium junceum*	[13]
***Kalmusia longispora***	*Dendrothyrium longisporum*	CBS 582.83 ^T^	JX496210	JX496097	JX496436	*Arceuthobium pusillum*	[15,16]
***Kalmusia longispora***	*Dendrothyrium longisporum*	CBS 824.84	JX496228	JX496115	JX496454	*Triticum aestivum*	[15,16]
***Kalmusia longispora***	*Dendrothyrium longisporum*	CBS 144250	MN945151	MN945157	MN939397	*Vitis vinifera*	This study
***Kalmusia sarothamni***		CBS 113833	KF796671	KF796675	nd	nd	[9]
***Kalmusia variispora***	*Dendrothyrium variisporum*	CBS 121517 ^T^	JX496143	JX496030	JX496369	*Vitis vinifera*	[15,16]
***Karstenula rhodostoma***		CBS 690.94	GU301821	nd	nd	nd	[16]
***Letendraea cordylinicola***		MFLUCC110148	KM213995	KM214001	nd	nd	[16]
***Letendraea cordylinicola***		MFLUCC110150	KM213996	KM214002	nd	nd	[16]
***Letendraea padouk***		CBS 485.70	AY849951	nd	nd	nd	[16]
***Montagnula aloes***		CBS 132531 ^T^	JX069847	JX069863	nd	nd	[16]
***Montagnula opulenta***		CBS 168.34	NG027581	nd	nd	nd	[16]
***Neokalmusia brevispora***	*Kalmusia brevispora*	KT 1466	AB524600	nd	nd	nd	[16]
***Neokalmusia scabrispora***	*Kalmusia scabrispora*	KT 2202	AB524453	nd	nd	nd	[16]
***Paraconiothyrium fuckelii***		CBS 764.71B	JX496225	JX496112	JX496451	Human	[15,16]
***Paraconiothyrium fuckelii***	*Coniothyrium rosarum*	CBS 508.94	JX496209	JX496096	JX496435	*Rosa* sp.	[15,16]
***Paraphaeosphaeria angularis***		CBS 167.70 ^T^	JX496160	JX496047	JX496386	*Saccharum officinarum*	[15,16]
***Paraphaeosphaeria arecacearum***		CBS 158.75 ^T^	JX496156	JX496043	JX496382	*Elaeis guineensis*	[15,16]
***Paraphaeosphaeria michotii***		CBS 340.86	JX496192	JX496079	JX496418	*Phragmites australis*	[15,16]
***Paraphaeosphaeria viridescens***		CBS 854.73 ^T^	JX681076	JX496085	JX496424	nd	[15,16]
***Phaeodothis winteri***		CBS 182.58	GU301857	nd	nd	nd	[16]
***Stagonospora paludosa***		CBS 135088	KF251760	KF251257	KF252740	nd	[16]

CBS, Westerdijk Fungal Biodiversity Institute, Utrecht, the Netherlands; MFLUCC, Mae Fah Luang University Culture Collection

^T^, the ex-type and ex-neotype strains, respectively; LSU: Large subunits of the nuclear ribosomal RNA gene; ITS, internal transcribed spacers and intervening 5.8S nrDNA; TUB, partial β-tubulin gene. nd: No data.

### Morphological observations

The isolate T15142 was grown on PDA, MEA (Sigma-Aldrich, Germany), and OA (Sigma-Aldrich, Germany) media, as well as on WA (agar from Sigma-Aldrich, Germany), at 25°C to examine its morphology. Conidia formation was induced by growing the fungus on sterilized pine needles placed on the surface of PDA medium at 25°C for 14 days [40]. The size of the conidia was defined from 100 measurements with ImageJ software [41]. Microscopic examinations were performed using an Alpha BIO-5f (Optika, Italy) microscope equipped with an Artcam-500MI camera (Artray, Japan).

### Pathogenicity tests

Pathogenicity tests were carried out using *V*. *vinifera* cv. Cabernet Sauvignon to evaluate the virulence of *K*. *longispora*. Shoot sections (with one node and one leaf), one year old potted cuttings and canes under field conditions were artificially infected. Cuttings were prepared by potting two-bud cane sections in a 1:1 mixture of perlite and commercial soil and were grown in a greenhouse. Green canes growing on trunks with cordon canopy management were used for the field studies. The grapevines were injured after surface sterilization with 70% v/v ethanol and inoculated with agar plugs (3 mm in diameter) containing actively growing mycelia of the *K*. *longispora* strains CBS 582.83 (ex-type culture), CBS 824.84, and T15142. Mock inoculations were set up by placing agar plugs without mycelia. All inoculations were performed on five grapevines for each strain. The shoots were placed in water and kept in a greenhouse as well as potted cuttings. Inoculated grapevines were subjected to 21 days (shoots), 80 days (cuttings) or 90 days (canes) of incubation before the examination of symptoms. Alongside the observation of developed symptoms, fungal strains were re-isolated from the vascular tissues. Discs were cut 5 mm above the inoculation points and cut into five pieces. These chips were surface-sterilized in sodium hypochlorite (4% available chlorine w/v), rinsed in 70% v/v ethanol, dried, and then placed on PDA medium. After incubation at 25°C for a week, the emerging mycelia were transferred to new plates and used for DNA extraction. The identity of the re-isolated fungi was verified by sequencing the ITS region.

### Examination of exoenzyme production

The secretion of digestive enzymes by the CBS 582.83, CBS 824.84, and T15142 *K*. *longispora* strains was compared. Minimal media (3% v/v glycerol, 0.15% w/v K_2_HPO_4_, 0.2% w/v KH_2_PO_4_, 0.1% w/v (NH_4_)_2_SO_4_, 0.5% w/v MgSO_4_, 0.2% w/v yeast extract, and 2% w/v agar) were prepared and supplemented with various substrates for the detection of different digestive enzymes. Ethyl cellulose (1% w/v) was used for cellulase, polygalacturonic acid (1% w/v) for pectinase, water-soluble starch (1% w/v) for amylase, and guaiacol (0.01% w/v) for laccase activity detection. The *K*. *longispora* strains were inoculated on these media as mycelial plugs of 3 mm in diameter growing on PDA medium. After 6–11 days of incubation at 25°C, the effects of the digestive enzymes were detected. Congo red staining was carried out for cellulase [42], precipitation with CTAB (hexadecyltrimethylammonium bromide) for pectinase [43], and Lugol’s staining for amylase [44] detection, while the visualization of laccase activity was based on the formation of a red-brownish reaction product from guaiacol incorporated into the medium [45].

Liquid cultures of the *K*. *longispora* strains were prepared for the quantification of exoenzyme activities. Strains were pre-grown on PDA medium for one week and mycelial plugs with 10 mm in diameter were cut from the margin of the colonies. One mycelial plug was inoculated into 50 ml of liquid minimal medium in a 100 ml Erlenmeyer flask and incubated in a rotary shaker (25°C, 180 rpm) for seven days. Sterile culture filtrates were obtained by the filtration of fermentation broths through a membrane with 0.45 μm pores. For the measurement of cellulase activity, 250 μl of culture filtrates were mixed with 250 μl cellulase assay solution (1%w/v carboxymethyl cellulose in 100 mM sodium citrate buffer pH 5.5) and incubated at 35°C for two hours. Reducing sugars were determined spectrophotometrically by the dinitrosalicylic acid method using glucose as a standard [46]. Cellulase activities were expressed as mg of released reducing sugar equivalent to glucose per hour. Pectinase activities were determined as described above in the case of cellulase activity, except that 1%w/v pectin was used in the assay solution instead of carboxymethyl cellulose and a 16 h incubation period was applied before the dinitrosalicylic acid assay. Laccase activity was measured using guaiacol as a substrate [47]. 500 μl of culture filtrates were mixed with 1500 μl of 10 mM guaiacol in 100 mM acetate buffer (pH 5.0) and the change of absorbance at 470 nm was monitored spectrophotometrically at room temperature for 10 mins. Laccase activities were expressed as ΔA_470_×min^-1^. Spectrophotometric measurements were done by the use of UV-1800 device (Shimadzu, Japan). All experiments were done in triplicates.

### Statistical analysis

Statistical comparisons were done by GraphPad Prism 5 software (GraphPad Software, San Diego California USA, www.graphpad.com) using One-way ANOVA with Tukey’s post-hoc test.

## Supporting information

S1 Table Individual values of cellulase, pectinase and laccase activities, measured in liquid cultures of strains CBS 824.84, CBS 582.83 and T15142 in three measurements. GE: Glucose equivalent.
(DOCX)
