# Supplementary material for: The fungus Kalmusia longispora is able to cause vascular necrosis on Vitis vinifera
Source: PLoS One. 2021 Oct 15;16(10):e0258043. doi: 10.1371/journal.pone.0258043 (PMC8519466; doi:10.1371/journal.pone.0258043)
Supplement: S1 Table — GE: Glucose equivalent. (DOCX) [file pone.0258043.s001.docx]

|  | **Cellulase (mg GE/h)** | **Pectinase (mg GE/h)** | **Laccase (ΔA_470_/min)** |  |
| --- | --- | --- | --- | --- |
| **CBS 824.84** | 0.285479 0.272616 0.299342 | 0.013203 0.012912 0.011923 | 0.0194 0.0214 0.022545 |  |
|  |  |  |  |  |
|  |  |  |  |  |
| **CBS 582.83** | 0.202192 0.189425 0.213959 | 0.014623 0.014726 0.014315 | 0.000637 0.00048 0.00068 |  |
|  |  |  |  |  |
|  |  |  |  |  |
| **T15142** | 0.152329 0.148589 0.156068 | 0.013334 0.012471 0.013493 | 0.0288 0.026 0.024787 |  |
|  |  |  |  |  |
|  |  |  |  |  |

**S1 Table**: Individual values of cellulase, pectinase and laccase activities, measured in liquid cultures of strains CBS 824.84, CBS 582.83 and T15142 in three measurements. GE: glucose equivalent
